# Cloning and expression analysis of a novel high-mobility group box 2 homologue from *Lampetra japonica*

**DOI:** 10.1007/s10695-013-9871-x

**Published:** 2013-10-25

**Authors:** Pang Yue, Xiao Rong, Xue Zhuang, Huang Jin Sha, Jin Min Li, Liu Xin, Qing Wei Li

**Affiliations:** Institute of Marine Genomics and Proteomics, Liaoning Normal University, Dalian, China

**Keywords:** *Lampetra japonica*, HMGB2, Proinflammatory

## Abstract

High-mobility group box 2 (HMGB2) is a nonhistone architectural protein that plays important roles in many biological processes. In this study, we cloned a homologue of the HMGB2 from the lymphocyte-like cells of *Lampetra japonica* (*L. japonica*). Sequence analysis reveals that *L. japonica* HMGB2 contains two highly conserved motifs and shares more than 70 % identity with the homologues from other vertebrate species. Subsequently, *Lj*-*HMGB2* was subcloned into the pET-28a(+) and pIRES2 AcGFP1-Nuc vector and expressed in Rosetta blue (DE3) and Hela cell lines, respectively. The recombinant *L. japonica* HMGB2 (rLj-HMGB2) with apparent molecular mass of 22 kDa was further purified by His-Bind affinity chromatography. Real-time quantitative PCR indicates that the expression level of Lj-HMGB2 was particularly up-regulated in intestines after challenged with lipopolysaccharide, while up-regulated in lymphocyte-like cells and heart after challenged with concanavalin A *in vivo.* In addition, rLj-HMGB2 could induce the generation of proinflammatory mediators in the activated human acute monocytic leukemia cell line (THP1), which suggested that Lj-HMGB2 may participate in the immune response of the lampreys.

## Introduction

High-mobility group box 2 (HMGB2) protein is a member of the HMGB protein family made up of two basic DNA-binding domains, HMG-box A and B, and a C-terminal acidic tail (Bustin 1999). Contrary to the ubiquitous expression of HMGB1, HMGB2 is restricted mainly to lymphoid organs and testes, although it is widely expressed during embryogenesis (Ronfani et al. 2001; Taniguchi et al. 2011). As nuclear proteins, HMGB2 is known to regulate various cellular activities, including transcription, DNA replication and repair (Bianchi and Agresti 2005). It binds to transcription factors such as Hox proteins (Zappavigna et al. 1996), steroid hormone receptors (Boonyaratanakornkit et al. 1998), p53 and p73 (Tros et al. 2002) and enhances the transcription and recombination activities of their partner proteins (Bianchi and Agresti 2005).

Despite the high degree of amino acid sequence similarity between HMGB1 and HMGB2, studies have shown that they have independent functions. Hmgb1−/− mice die shortly after birth because of hypoglycemia (Calogero et al. 1999; Rovere-Querini et al. 2004), while hmgb2−/− mice are viable, but male mice have reduced fertility, as HMGB2 seems to play a role in germ cell differentiation (Ronfani et al. 2001). Recently, studies demonstrated a correlation between HMGB2 expression and progenitor maintenance in the superficial zone (SZ) (Taniguchi et al. 2009a, b). It was reported that HMGB2 can be secreted by THP-1 cells and promotes proliferation and migration of endothelial cells; in addition, it is also an important cytokine (Pusterla et al. 2009).

Lampreys are considered to be one of the most ancient vertebrates still living today, which are an important species between invertebrates and vertebrates; therefore, it will mark an evolutionary history in the aspects of genetic information. In addition, as the direct ancestor of vertebrates, it will provide abundant genetic information base for vertebrate origin and evolution. Nikitina et al. put forward that the lamprey is an ideal animal model to study the vertebrate evolution, embryo development and the origin of adaptive immune system (Sower et al. 2006; Amemiya et al. 2007). Besides, the parasitic lampreys are also known as blood suckers in the marine which usually use their oral disks to suck the blood and body fluids of the host fishes (Rovainen 1996; Gross and Manzon 2011). Recently, more and more reports have been focused on the protein with important physiological functions, such as anesthesia, anti-coagulation and vasodilation, from the buccal gland, liver or lymphocyte-like cells of the adult lampreys (Ito et al. 2007; Xiao et al. 2007; Sun et al. 2008; Chi et al. 2009).

In contrast to the extensive studies of HMGB2 in vertebrates, little is known about the biological activities and physiological roles of HMGB2 in the jawless lampreys. In the present study, we report for the first time on the molecular cloning and characterization of a HMGB2 homologue from *Lampetra japonica* by analyzing the expressed sequence tags (ESTs) of the cDNA library of lymphocyte-like cells. Our results suggest that the *L. japonica* HMGB2 (Lj-HMGB2) is a highly conserved protein and is widely distributed in the gill, intestine, lymphocyte-like cells, heart and kidney of adult lampreys. Interestingly, the expression level of HMGB2 in the heart, intestine and lymphocyte-like cells is increased significantly after the lampreys are challenged by LPS or concanavalin A (ConA). In addition, proinflammatory activity of the recombinant *L. japonica* HMGB2 is also discussed.

## Materials and methods

### Materials

The handling of lamprey and all experimental procedures were approved by the Animal Welfare and Research Ethics Committee of the Institute of Dalian Medical University (permit number SYXK2004-0029). Adult lampreys (*L. japonica*) were obtained in December from the Tongjiang Valley of Songhua River in Heilongjiang Province in China. Peripheral blood was collected from the caudal subcutaneous sinus of lampreys, and leukocytes were extracted by Percoll gradient centrifugation. Lamprey leukocytes were sorted into three discrete subpopulation based on the forward and sideward light-scattering profiles from flow cytometry. Lymphocyte-like cells were collected from a subpopulation using FACSAria II (BD Biosciences). The cDNA library construction of the lymphocyte-like cells and the ESTs sequencing had been completed by our laboratory.

### Identification and cloning of HMGB2 homologue from lymphocyte-like cells of *L. japonica*

Based on the analysis of the ESTs, a part-length HMGB2 homologue was identified in the ESTs of the lymphocyte-like cells from *L. japonica* using NCBI’s Basic Local Alignment Search Tool (BLAST). Total RNA was isolated from lymphocyte-like cells of *L. japonica* using Trizol (GIBCO BRL), and cDNA was obtained according to the manufacturer’s instruction of High Fidelity PrimeScript™ RT-PCR Kit (TaKaRa). PCR amplification of a full-length HMGB2 was performed by 5′ Full RACE kit (TaKaRa). The two reverse primers (5′-CCCAGCCGTTTGGCAATCTCACC-3′ and 5′-GGTGCATTAGGGTCCTTTGTCTT-3′) were designed on the basis of the EST homologous to the HMGB2. The amplified product was then purified and cloned into pMD19-T vector using DNA Ligation kit (TaKaRa, China) and transformed into *Escherichia coli* strain DH5α as the host bacterium. DNA sequencing was conducted with M13 forward/reverse primers using a model 377 DNA sequencer (ABI 100).

### Real-time quantitative PCR analysis of the expression pattern of Lj-HMGB2

Adult lampreys in blank group (*N* = 3), LPS challenged group (*N* = 3) and ConA challenged group (*N* = 3) were intraperitoneally injected with PBS, LPS (10 μg) and ConA (10 μg), respectively. After 24 h, total RNA was extracted from gills, heart, intestines, lymphocyte-like cells and kidneys using Trizol (GIBCO BRL) in accordance with the manufacture’s instructions and treated with DNase I (QIAGENCat: no. 79254) to remove contaminated DNA. Reverse transcription was performed as described previously (Liu et al. 2009). The real-time quantitative PCR was carried out with the TaKaRa SYBR^®^ PrimeScript™ RT-PCR Kit according to the manufacturer’s protocol. Each reaction contained the following: 12.5 μl SYBR *Premix Ex Taq* (2×), 1 μl of each primer (10 μM), 2 μl cDNA and water to a final volume of 25 μl. The amplification was carried out on TaKaRa PCR Thermal Cycler Dice Real Time System with the parameters as follows: initial denaturation at 95 °C for 10 s to activate DNA polymerase followed by 45 cycles of 5 s at 95 °C, 30 s at 60 °C and 30 s at 72 °C. Specific primers for Lj-HMGB2 were 5′-CTACTCACGCCCGAGACTAAATC-3′ (forward) and 5′-CCGCCACGTCCTTCTCAT-3′ (reverse). Glyceraldehyde-3-phosphate dehydrogenase (GAPDH, GenBank number AY578058) was utilized as an internal control, and its primers were 5′-AACCAACTGCCTGGCTCCT-3′ (forward) and 5′-GTCTTCTGCGTTGCCGTGT-3′ (reverse). Each sample was analyzed in triplicate. Data were analyzed with the Thermal Cycler Dice Real Time System analysis software (TaKaRa).

### Expression vector construction

The open reading frame (ORF) of Lj-HMGB2, the forward primer (5′-AAATGGGTCGCGGATCCGAATTCATGGGTAAAGGAGAGCCAGG-3′) incorporated a *Eco*R I site (underlined), whereas the reverse primer (5′-GGTGCTCGAGTGCGGCCGCCTACTCGTCATCATCCTCATC-3′) incorporated a stop codon (TAG) and a *Not* I site (underlined), was amplified and subcloned into the pET-28a(+) vector with the His-tag. The correct recombinant prokaryotic expression vector was named as pET-28a(+)-LjHMGB2.

pET-28a(+)-LjHMGB2 recombinant plasmid was enzymed by *Not* I, blunted by DNA blunting kit (TaKaRa, China) and then enzymed by *Eco*R I. The purified product was inserted into the corresponding region of pIRES2-AcGFP1-Nuc expression vector (*Eco*R I/*Sma* I). Positive clones were first selected by PCR and reconfirmed by restriction digestion and sequencing. The correct recombinant eukaryotic expression vector was named as pIRES2-AcGFP1-Nuc-LjHMGB2.

### Expression and purification of recombinant Lj-HMGB2 in vitro

The recombinant Lj-HMGB2 was expressed in *Rosetta blue* induced by 1 mM isopropyl-1-thio-β-d-galactopyranoside (IPTG). Subsequently, the cells were collected via centrifugation, washed and resuspended in 20 mM Tris–HCl buffer containing 1 mM EDTA (pH 8.0). The cell suspension was sonicated for 30 min on ice and centrifuged again at 14,000 rpm for 20 min at 4 °C. The soluble supernatant was collected and subjected to a Ni–NTA His-Bind resin column (Novagen) equilibrated with PBS. After washing the column with PBS, the recombinant protein was collected in elution buffer with 50 mM Tris–HCl (pH 8.0). The concentration of rLj-HMGB2 was measured using a bicinchoninic acid (BCA) Protein Assay kit (BEYOTIME) according to the manufacture’s instructions. The purified rLj-HMGB2 was analyzed by 12 % SDS–PAGE by the method of Laemmli (Laemmli 1970). The proteins were stored at −80 °C until used.

### Preparation of anti-lamprey HMGB2 polyclonal antibody and Western blots

Production of anti-rLj-HMGB2 antibody followed the procedures described by our previous report (Pang et al. 2012). The antibody titer was determined by enzyme-linked immunosorbent assay (ELISA). The antibody specificity was confirmed by Western blotting using the recombinant Lj-HMGB2 protein, human HMGB1 (Sigma) and *L. japonica* lymphocyte-like lysate.

### Measurement of TNF-α by ELISA

To determine the production of inflammatory mediators such as TNF-α in the activated macrophages, THP-1 cells were cultured in RPMI-1640 medium (Invitrogen, Carlsbad, CA, USA) containing 10 % fetal bovine serum, 2 mM glutamine and 1 % streptomycin/penicillin and stimulated with an irrelevant His-tagged protein (50 ng/ml), rLj-HMGB2 (50 ng/ml), rLj-HMGB1 (50 ng/ml), LPS (50 ng/ml) and rHu-HMGB1 (50 ng/ml). The culture supernatants were collected at 0, 6, 12, 24 and 48 h poststimulation and the levels of TNF-α were measured using ELISA kits, according to the manufacturer’s instructions (Boster. Biological, Technology, Ltd.). The samples were assayed in triplicate, and each experiment was repeated at least three times.

### Cell culture and transfection

Hela cells were maintained in RPMI1640 (Invitrogen, Carlsbad, CA, USA) supplemented with 1 % l-glutamine, 100 U/ml penicillin, 100 μg/ml streptomycin and 10 % heat-inactivated fetal calf serum and grown in humidified 5 % CO_2_ at 37 °C. In 12-well plate, the optimal cell number is 2 × 10^5^/well. The cells were seeded at 24 h before gene transfection; 2 × 10^5^ cells were transfected by 2.5 μg pIRES2-AcGFP1-Nuc-LjHMGB2 plasmid DNA with 0.75 μl Xfect™ transfection reagent (Clontech, USA), according to the manufacture’s protocol. Control Hela cells were transfected with pIRES2-AcGFP1-Nuc or no plasmid DNA. Fluorescence imaging was performed 24 h after transfection on an Olympus IX-71 (Olympus IX71, Olympus Optical Co. Ltd., Tokyo, Japan).

### Lj-HMGB2 mRNA expression analysis after transfection

Total RNA was isolated from Hela cells or transfectants using Trizol (GIBCO BRL), and cDNA was obtained according to the manufacturer’s instruction of High Fidelity PrimeScript™ RT-PCR Kit (TaKaRa). Primer used was the same as real-time PCR primer. The amplification of Lj-HMGB2 gene was carried out with the *TaKaRa EX Taq* Kit according to the manufacturer’s protocol.

### Statistical analysis

All data were presented as mean ± SE based on separate experiments. Student’s *t* test was used for determining statistical significance.

## Results

### Molecular cloning of Lj-HMGB2 from lymphocyte-like cells of *L. japonica*

**Fig. 1:**
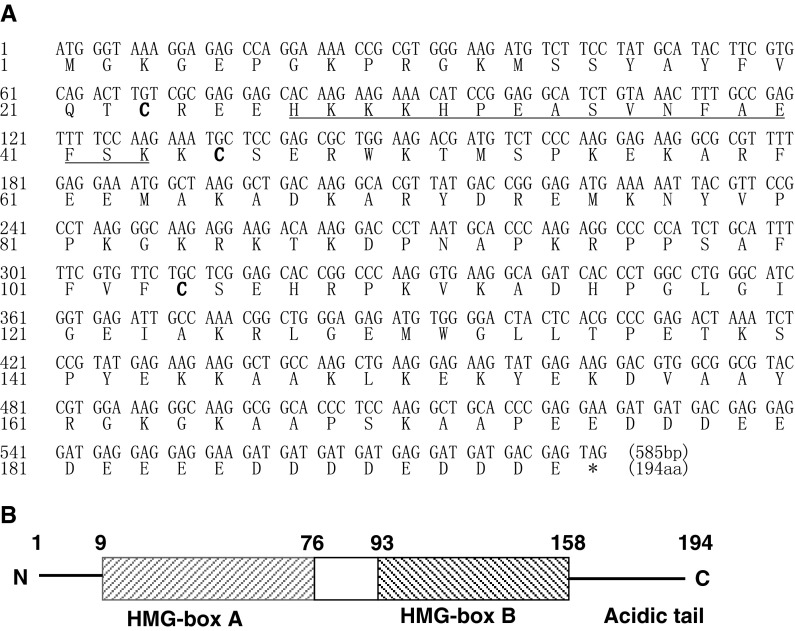
ORF and structural schematic of Lj-HMGB2. Nucleotide and deduced amino acid sequences of lamprey HMGB2 ORF (**a**). Nucleotide (*upper line*) and amino acid (*lower line*) sequences are numbered from the initiation of methionine. Stop codon is marked with an *asterisk*. Peptides corresponding to the *single underlined* sequence (His_27_–Lys_43_) are lysine-rich nuclear localization sequences (NLSs). Three cysteine (Cys_23_, Cys_45_ and Cys_104_) residues appear in *bold*. Structural schematic of Lj-HMGB2 domains (**b**)

The full-length cDNA of Lj-HMGB2 was obtained from cDNA library of lymphocyte-like cells from *L. japonica*, which was a 5′-untranslated region (UTR) of 167 bp and an ORF of 585 bp encoding a polypeptide of 194 amino acids with an estimated molecular mass of 22.2 kDa and a theoretical isoelectric point of 6.93 (Fig. 1a) (ProtParam program of ExPASy, http://www.expasy.ch/tools/protparam.html). In addition, Lj-HMGB2 contains a positively charged amino acid sequence segment (His_27_–Lys_43_), called lysine-rich nuclear localization sequences (NLSs), and three cysteines (Cys_23_, Cys_45_ and Cys_104_). The nucleotide sequence of *Lj*-*HMGB2* has been submitted to GenBank database with the accession number of HQ615992. Sequence analysis shows that the Lj-HMGB2 also possesses an N-terminal HMG-box A domain (Pro_9_–Lys_76_), a central HMG-box B domain (Pro_93_–Ala_158_), a C-terminal acidic tail (Glu_174_–Glu_194_) and a linker (Asn_77_–Pro_93_) (Fig. 1b) (MEME Suite Motif-based sequence analysis tools, http://meme.sdsc.edu/meme/intro.html).

### The expression pattern of Lj-HMGB2 in various tissues of *L. japonica* after LPS or ConA challenge

**Fig. 2:**
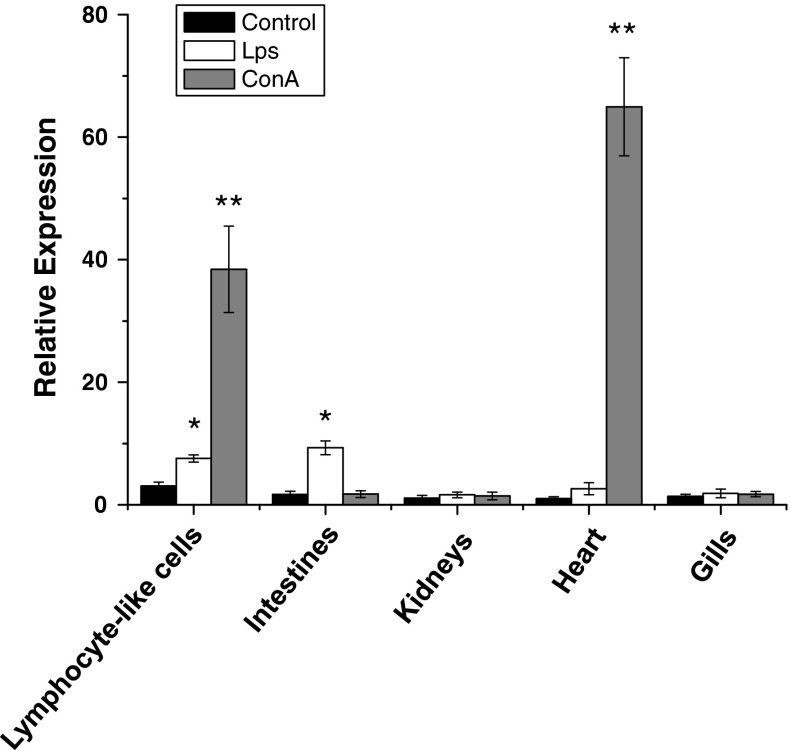
The expression level of Lj-HMGB2 in different tissues from adult lampreys after LPS or ConA challenge. Real-time quantitative RT-PCR was done with RNA samples from gills, intestines, lymphocyte-like cells, heart and kidneys of *L. japonica* stimulated with PBS, LPS and ConA. The lamprey GAPDH was used as an internal control to calibrate the cDNA template for all the samples. The significant differences (*P* < 0.05) in HMGB2 expression between the challenged groups and the blank group were indicated with *asterisks* (**P < 0.01)

To determine the tissue distribution of Lj-HMGB2, real-time quantitative PCR analysis was performed in heart, gills, intestines, lymphocyte-like cells and kidneys of *L. japonica* after LPS or ConA challenge in vivo with the GAPDH as an internal control. Compared to negative controls stimulated by PBS, mRNA expression of *Lj*-*HMGB2* was up-regulated significantly in heart (60-fold increase) and in lymphocyte-like cells (12-fold increase) in ConA-group challenged animals 24 h postinjection, while the expression level of Lj-HMGB2 was up-regulated in intestines (fivefold increase) in LPS group (Fig. 2).

### Expression of recombinant Lj-HMGB2 and production of antibodies

**Fig. 3:**
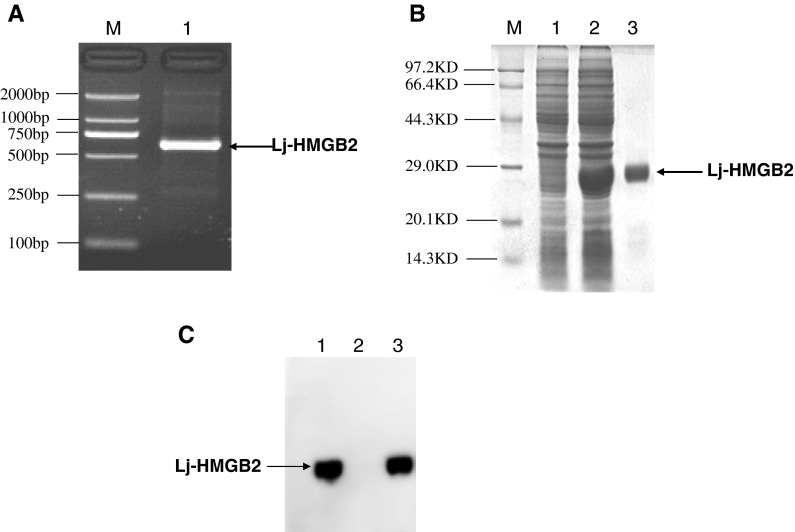
Lj-HMGB2 gene amplification, rLj-HMGB1 purification and Western blotting. **a** Amplification of Lj-HMGB2 gene fragment by PCR. **b** Expression and purification of recombinant six-His-tagged Lj-HMGB2 proteins. *Lane M* low molecular weight protein marker, *lane 1* crude lysate of *E. coli* before induction, *lane 2* lysate of *E. coli* transfected by Pet28a(+)-Lj-HMGB2 expression vector, *lane 3* purified recombinant Lj-HMGB2 protein. **c** Western blotting analysis of Lj-HMGB2 by rabbit anti-Lj-HMGB2 polyclonal antibodies. *Lane 1* *L. japonica* lymphocyte-like lysate, *lane 2* human HMGB1 protein, *lane 3* recombinant Lj-HMGB2 protein

As shown in Fig. 3a, Lj-HMGB2 fragment of 588 bp in length, consistent with the expected size, was cloned by PCR. The purified PCR product was digested with *Eco*R I/*Not* I and cloned into the pET-28a(+) vector (5,369 bp) treated with the corresponding enzymes. Recombinant lamprey HMGB2 protein (rLj-HMGB2) was expressed as a histidine tag fusion protein in *Rosetta blue*. The purified rLj-HMGB2 migrated as a single band on a 12 % SDS–PAGE gel with a molecular mass of about 26 kDa (Fig. 3b). BCA assay shows that the concentration of purified rLj-HMGB2 was about 0.6 mg/ml. Rabbit polyclonal antibodies against rLj-HMGB2 were purified by protein G Sepharose and confirmed by Western blotting. A 26-kDa band corresponding to the recombinant proteins and a 22-kDa band corresponding to the native rLj-HMGB2 proteins from lymphocyte-like cells were recognized by anti-Lj-HMGB2 antibodies.

### rLj-HMGB2 induces the production of TNF-α in THP-1 monocytic cells

**Fig. 4:**
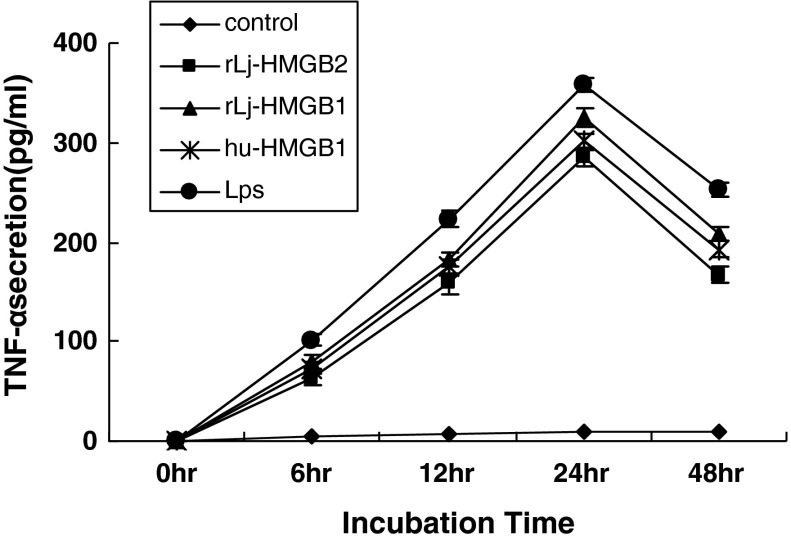
Lamprey HMGB2 induced TNF-α production in THP-1 monocytic cells. THP-1 cells (1 × 10^9^ cell/l) were incubated with rLj-HMGB2, rLj-HMGB1 (50 ng/ml), LPS (50 ng/ml), rLj-HBP1 (50 ng/ml) or human HMGB1 (50 ng/ml) for various lengths of time. Human HMGB1 and LPS were used as a positive control, and rLj-HBP1 was used as a negative control. The concentration of released TNF-α in the culture supernatants was measured by ELISA

Andersson and Tracey (2011) reported that human HMGB proteins induced TNF-alpha production from monocyte cells, and therefore, we wanted to determine whether lamprey HMGB2 can stimulate proinflammatory cytokines in monocytes. rLj-HMGB2 (50 ng/ml) significantly increased the secretion of TNF-α in THP-1 monocytic cells in a time-dependent manner (from 0 to 24 h). And the largest secretion of TNF-α was detected at 24 h, almost 3 times higher than that of control group. After that, the release of TNF-α began to decrease and reached 50 % of the maximum value at 48 h. We compared the amount of TNF-α released in the presence of Lj-HMGB2 to that released when the cells were exposed to Hu-HMGB1, rLj-HMGB1 or LPS and found that similar amounts of TNF-α were released under all conditions. To ensure that contaminants from the purification process were not responsible for the release of TNF-α, we tested a related, but irrelevant, His-tagged protein (Lj-HBP1). Treatment with Lj-HBP1 did not cause a release of TNF-α (Fig. 4). Our results suggest that rLj-HMGB1 stimulates THP-1 monocytic cells to release proinflammatory cytokines.

### Establishment of transfectants of Hela cells that overexpress Lj-HMGB2

**Fig. 5:**
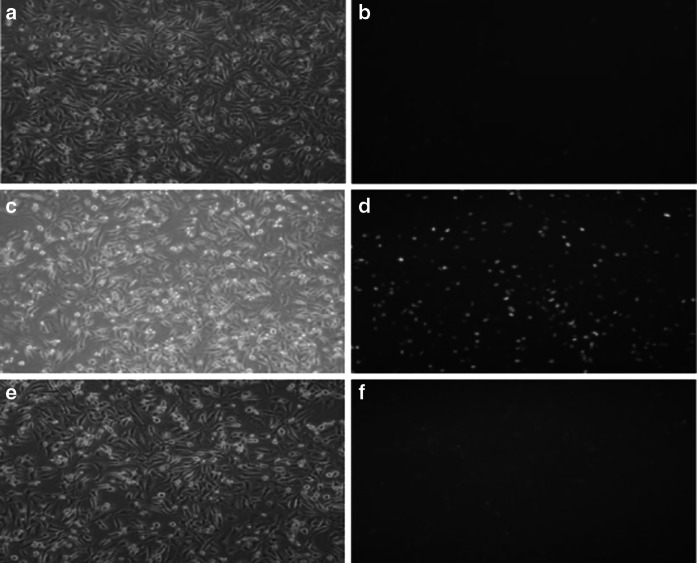
GFP gene transfection results in Hela cells (10 × 10). GFP expression mediated by pIRES2-AcGFP1-Nuc plasmid (**b**), pIRES2-AcGFP1-Nuc-LjHMGB2 plasmid (**d**), Hela cells with no plasmid transfected (used as negative control) (**f**), the corresponding images under bright field (**a**, **c**, **e**)

**Fig. 6:**
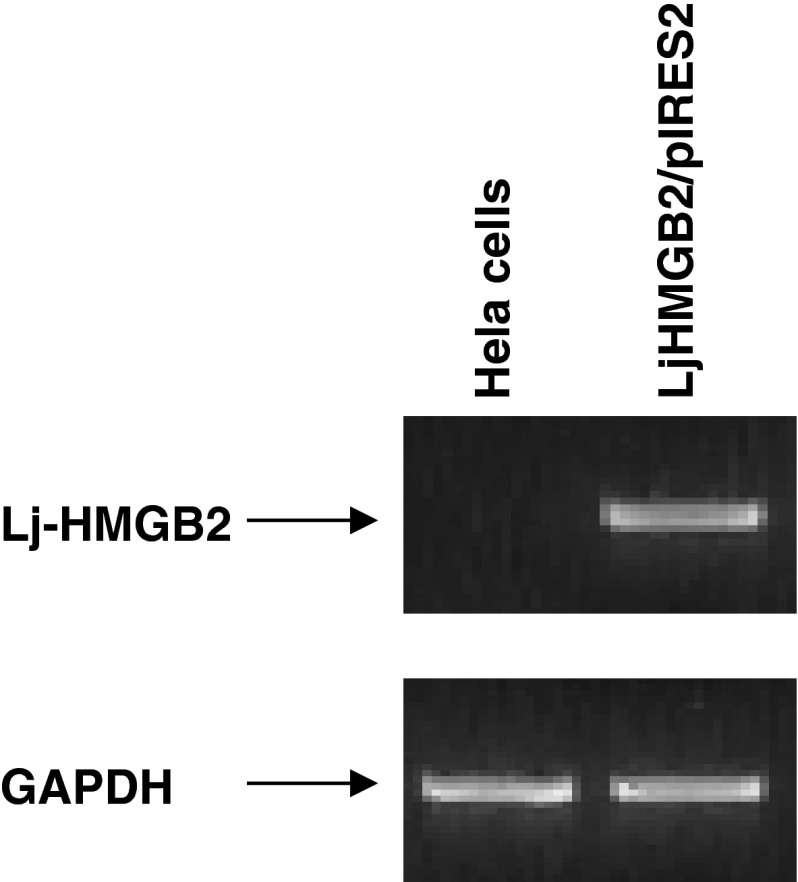
Lj-HMGB2 mRNA expression in transfected Hela cells. PCR showing levels of Lj-HMGB2 and GAPDH mRNA expression in transfected Hela cells and in untransfected Hela cells

We prepared three sets of parallel cultures of Hela cells, as described in “Materials and methods” section: (1) cells transfected with pIRES2-AcGFP1-Nuc, (2) cells transfected with pIRES2-AcGFP1-Nuc-LjHMGB2 and (3) untransfected Hela cells. Figure 5 shows diagrams of green fluorescent protein (GFP) expression. GFP levels from cells transfected with pIRES2-AcGFP1-Nuc-LjHMGB2 were greatest. Figure 6 illustrates mRNA expression data showing Lj-HMGB2 mRNA levels from Lj-HMGB2-transfected cells and untransfected Hela cells. Lj-HMGB2 mRNA expression was greatest from Lj-HMGB2-transfected cells. Expression of the GAPDH mRNA was essentially identical in all Hela cells.

## Discussion

In our previous work, we had identified high-mobility group box 1 gene from *L. japonica* and defined the biological function (Pang et al. 2012). In this study, we isolated a homologue of HMGB2 gene and demonstrated that the lamprey HMGB2 shares over 70 % identities to human HMGB1, HMGB2 and HMGB3. However, Moleri et al. (2011) previously suggested that HMGB of jawless fish (lamprey and hagfish) has diverged from a common ancestral gene of fish HMGB1–3. In addition, Sharman et al. (1997) isolated HMG1 cDNA clone from *Lampetra fluviatilis*, and their phylogenetic analysis indicates that lamprey HMG1 diverged from the mammalian HMGB genes before mammalian HMGB split into HMGB1 and HMGB2 subfamilies. However, we have cloned the homologue of HMGB2 gene from the cDNA library of lymphocyte-like cells in Japanese lamprey (*L. japonica*). Phylogenetic analysis and domain/motif analysis revealed that Lj-HMGB2 was grouped to HMGB2 gene cluster of jawed fish (Pang et al. 2012); in addition, lamprey HMGB2 falls outside the vertebrate clade before HMGB split into HMGB1, HMGB2 and HMGB3 subfamilies.

Sequence analysis shows that the Lj-HMGB2 contains a positively charged amino acid sequence segment and three cysteines (Fig. 1a). Human HMGB1 (GenBank number P09429) contains three cysteines (Cys_23_, Cys_45_ and Cys_106_), which can form a Cys_23_–Cys_45_ disulfide bond in the setting of oxidative stress (Tang et al. 2010). The proinflammatory activity domain of HMGB1 is localized in the first 20 amino acids of the HMG-box B (corresponding to HMGB1 amino acid residues 89–108), which has the most significant cytokine functionality. HMGB1 Cys_106_ is required for TLR4 binding and cytokine release from macrophages (Li et al. 2013). In addition, Lj-HMGB2 also possesses an N-terminal HMG-box A domain, a central HMG-box B domain, a C-terminal acidic tail and a linker (Fig. 1b). These findings suggest that the Lj-HMGB2 molecule has biological function, although the number of amino acid residues is different from mammalian HMGB2. Furthermore, our studies involved the production of recombinant Lj-HMGB2 protein and investigation of its biochemical roles. The present study demonstrated that lamprey HMGB2 protein induced the release of TNF-α from THP1 cells (Fig. 4), indicating that Lj-HMGB2 is capable of regulating the mammalian innate immune system that defends animals from the invasion of pathogens and other exogenous injuries to a similar extent as Hu-HMGB1/2.

Real-time PCR analysis indicated that mRNA expression of Lj-HMGB2 was up-regulated significantly in hearts and lymphocyte-like cells in ConA-group challenged animals after 24 h postinjection, while the expression level of Lj-HMGB2 was up-regulated in intestines and lymphocyte-like cells in LPS group (Fig. 3). The fact that LPS/ConA up-regulates Lj-HMGB2 expression suggests that Lj-HMGB2 triggers the inflammatory response in the lamprey hearts, lymphocyte-like cells and intestines. Our research group had reported several mRNA expression results by real-time PCR, such as CD29 (Wu et al. 2010) and CD9 (Wu et al. 2012). After the stimulations with LPS/ConA, these mRNA expression levels increased significantly in the heart. Previous studies have proved that mammal HMGB2 was present in the thymus, testes and lymphoid tissues of adult mice and was also expressed (Ronfani et al. 2001). Moreover, in our previous work, we cultured cells from the heart of the lamprey and found that the heart tissue contained lymphoid-like cells. So, we analyze that the heart of lamprey expressed considerable HMGB2 and might be another hemopoietic tissue, whereas the intestine of cyclostomes has been shown to be an active hemopoietic tissue (Mayer et al. 2002; Rombout et al. 2005; Suzuki et al. 2005).

In addition, Lj-HMGB2 gene was successfully constructed to pIRES2-AcGFP1-Nuc eukaryotic expression vector, then the recombinant plasmid pIRES2-AcGFP1-Nuc-Lj-HMGB2 was transfected to Hela cells, and GFP was expressed under fluorescence microscopy. Eukaryotic expression vector of lamprey HMGB2 gene was constructed and transfected, which lay foundation for the lamprey HMGB2 gene function research in lamprey and other related studies.

In conclusion, this report represents for the first time the molecular cloning and characterization of HMGB2 from *L. japonica*. We discovered that HMGB2 acts as cytokine in lamprey. Thus far, a few studies have been carried out on agnathan cytokines, such as IL-8 (Najakshin et al. 1999), IL-17 (Tsutsui et al. 2007) and MIF (Sato et al. 2003). Our finding suggests the possible presence of a complicated and sophisticated cytokine network in agnathan. Future studies will aim to identify more cytokines and reveal their functions and interaction in the biodefense system in lamprey.

## Abbreviations

BCA: Bicinchoninic acid
ConA: Concanavalin A
EST: Expressed sequence tag
GAPDH: Glyceraldehyde-3-phosphate dehydrogenase
HMGB2: High-mobility group box 2
IPTG: Isopropyl-1-thio-β-d-galactopyranoside
*L. japonica*: *Lampetra japonica*
Lj-HMGB2: *L. japonica* HMGB2
LPS: Lipopolysaccharide
ORF: Open reading frame
rLj-HMGB2: Recombinant *L. japonica* HMGB2
THP1: Human acute monocytic leukemia cell line
